# Antitumour effect of the mitochondrial complex III inhibitor Atovaquone in combination with anti-PD-L1 therapy in mouse cancer models

**DOI:** 10.1038/s41419-023-06405-8

**Published:** 2024-01-11

**Authors:** Gonzalo Rodriguez-Berriguete, Rathi Puliyadi, Nicole Machado, Alessandro Barberis, Remko Prevo, Martin McLaughlin, Francesca M. Buffa, Kevin J. Harrington, Geoff S. Higgins

**Affiliations:** 1 Department of Oncology, University of Oxford, Oxford, UK; 2 Nuffield Department of Surgical Sciences, University of Oxford, Oxford, UK; 3 Institute of Cancer Research, London, UK; 4 Department of Computing Sciences, Bocconi University, Milan, Italy

**Keywords:** Cancer metabolism, Cancer

## Abstract

Immune checkpoint blockade (ICB) provides effective and durable responses for several tumour types by unleashing an immune response directed against cancer cells. However, a substantial number of patients treated with ICB develop relapse or do not respond, which has been partly attributed to the immune-suppressive effect of tumour hypoxia. We have previously demonstrated that the mitochondrial complex III inhibitor atovaquone alleviates tumour hypoxia both in human xenografts and in cancer patients by decreasing oxygen consumption and consequently increasing oxygen availability in the tumour. Here, we show that atovaquone alleviates hypoxia and synergises with the ICB antibody anti-PD-L1, significantly improving the rates of tumour eradication in the syngeneic CT26 model of colorectal cancer. The synergistic effect between atovaquone and anti-PD-L1 relied on CD8+ T cells, resulted in the establishment of a tumour-specific memory immune response, and was not associated with any toxicity. We also tested atovaquone in combination with anti-PD-L1 in the LLC (lung) and MC38 (colorectal) cancer syngeneic models but, despite causing a considerable reduction in tumour hypoxia, atovaquone did not add any therapeutic benefit to ICB in these models. These results suggest that atovaquone has the potential to improve the outcomes of patients treated with ICB, but predictive biomarkers are required to identify individuals likely to benefit from this intervention.

## Introduction

During the last decade, the introduction of therapies targeting immune checkpoints (immune checkpoint blockade, ICB) has profoundly changed the landscape of cancer treatment, providing long-lasting responses in several cancer types. Antibodies against the T cell plasma membrane receptors Cytotoxic T-lymphocyte-associated protein 4 (CTLA-4) and programmed cell death 1 (PD-1), or its ligand PD-L1, are the most widely used ICB therapies clinically. Mechanistically, ICB blocks inhibitory signalling through these receptors that would otherwise attenuate T cell responses, which results in the unleashing of a robust anti-tumour immune response. Despite these successes, most patients treated with ICB experience relapse or do not respond at all, prompting the need for novel therapeutic strategies to enhance their efficacy [1].

Tumour hypoxia, a frequent feature of solid tumours, is thought to be a key factor limiting ICB efficacy, by repressing the cytotoxic activity of NK and T cells, promoting the activity and tumour recruitment of immunosuppressive cells (e.g., myeloid-derived suppressor cells, Tregs), and favouring the establishment of an immunosuppressive tumour microenvironment [2]. Increasing tumour oxygen levels by either supplemental oxygenation or treatment with a hypoxia-activated pro-drug has been shown to improve the response to anti-PD-1 and anti-CTLA-4 in cancer models, through undefined mechanisms linked to enhanced CD8+ T cell-mediated immunity [3, 4]. Although encouraging, these approaches for hypoxia alleviation are difficult to translate to clinic, because of either practical issues or lack of data on efficacy and toxicity in humans.

Reducing the oxygen consumption of tumour cells has been proposed as an effective strategy to alleviate tumour hypoxia [5–7]. This can be achieved by inhibiting oxidative phosphorylation (OXPHOS), the metabolic pathway that uses the mitochondrial electron transport chain (ETC) coupled to oxygen consumption to generate energy. We found that the antimalarial drug atovaquone reduces oxygen consumption in vitro by inhibiting the ETC Complex III and decreases tumour hypoxia in xenograft mouse models [8]. Recently, our group completed a Phase I clinical trial (ATOM, NCT02628080) which showed that atovaquone safely alleviates tumour hypoxia in non-small cell lung cancer patients [9, 10]. Considering the immune-suppressive role of hypoxia and the promising prospect of using atovaquone as a hypoxia modifier in a clinical scenario, we asked whether atovaquone could add therapeutic benefit to ICB. In the present study, we evaluated the efficacy and safety of atovaquone plus anti-PD-L1 (aPD-L1) in vivo and investigated the implication of the anti-tumour immunity in this combination treatment.

## Materials and methods

### Cell lines and spheroids

Mouse CT26 (colorectal), 4T1 (breast), LLC (lung) and MC38 (colorectal) cancer cells were obtained from the American Type Culture Collection (ATCC). These cell lines were cultured in RPMI-1640 (CT26, 4T1) or DMEM (LLC, MC38) medium (Merck) supplemented with 10% FBS, at 37°C and 5% CO_2_, and were routinely tested for mycoplasma using MycoAlert (Lonza). Cell line authentication was performed by short tandem repeat profiling by LGC Standards. Spheroids were generated by seeding CT26 single-cell suspensions (3 × 10^5^ cells/well) in 96-well ultra-low attachment U-bottom plates (Costar). For the in vitro experiments, atovaquone (Sigma-Merck) was dissolved in DMSO. Treatments were started 3 days after seeding, at an approximate spheroid diameter of 450–500 µm. Spheroid diameter was measured on brightfield images acquired with a GelCount scanner (Oxford Optotronix).

### Hypoxia assessment

For hypoxia quantification we used EF5 (Merck), a compound that specifically binds to hypoxic cells. In the experiments with spheroids, 200 µM EF5 was added 6 h before the end of treatments. In the in vivo experiments, 0.01 mL/g body weight of 10 mM EF5 dissolved in PBS was administered by intraperitoneal (i.p.) injection 4 h before culling. Then, the tumours/spheroids were fixed with 4% paraformaldehyde for 6 h at 4 ^o^C, and transferred to 30% sucrose at 4 ^o^C overnight. Samples were embedded in OCT matrix (WVR) and frozen on dry ice, and 5 µm (spheroids) or 10 µm (tumours) sections were prepared using a Leica CM1860 cryostat. Middle-plane spheroid sections were selected for staining with the anti-EF5-Alexa Fluor 488 antibody (clone ELK3-51, #EF5010, Merck), as described previously [8]. Images were acquired using a Nikon NiE fluorescence microscope and analysed with Imaris software (Oxford Instruments).

### Oxygen consumption rate (OCR) and extracellular acidification rate (ECAR) assessment

Cells were seeded suspended in RPMI/10% FBS medium in 96-well Seahorse assay plates and left overnight to attach in the incubator. Then, the medium was replaced with XF assay medium (Seahorse Biosciences) containing 5 mM glucose, 5 mM sodium pyruvate and 4mM L-glutamine, and OCR and ECAR were simultaneously measured in real-time using an XF96 Analyzer (Seahorse Biosciences), before and after the addition of the different treatments. Just after the OCR/ECAR measurement, cells were fixed with ice-cold 70% ethanol, stained with 1 µg/mL Hoechst, and imaged and counted using a Celigo Image Cytometer (Nexcelom Bioscience).

### Animals and tumour growth delay assay

The project licence covering the animal work (PP5787245) was approved by the Animal Welfare and Ethical Review Body (AWERB) (University of Oxford), and granted by the UK Home Office Animals in Science Regulation Unit (ASRU) under the Animals (Scientific Procedures) Act 1986 (ASPA). Sample size was estimated based on previous pilot studies. Tumours were induced by a subcutaneous injection of CT26 (4 × 10^5^ cells), LLC (2.5 × 10^5^) or MC38 (2.5 × 10^5^) cells, suspended in 30% Matrigel (Corning) diluted in PBS, on the right flank of 6–7-week-old female Balb/c (CT26) or C57/BL6 (LLC, MC38) mice. The day after, mice were randomly allocated to the different treatment groups using a randomiser (random.org). Treatments were started when tumours reached 25–40 mm^3^. Atovaquone (Wellvone, GSK, 200 mg/kg/day) or vehicle alone (0.1% carboxymethyl cellulose (CMC)) were administered at single daily doses by oral gavage. Rat anti-mouse PD-L1 (clone 10 F.9G2, #BE0101) or the corresponding isotype control (clone LTF-2, #BE0090) were administered via i.p. injection, at a dose of 10 mg/kg every 3 days for a total of 5 doses. Rat anti-mouse CD8α (aCD8; clone 2.43, # BE0061) or the corresponding isotype control (clone LTF-2) were administered by i.p. injection, at a dose of 400 µg 2 days before and 1 day after the first aPD-L1/atovaquone dose, and 200 µg every 6 days thereafter. Complete depletion of CD8+T cells was confirmed by FACS in blood sampled 3 and 14 days after the first aCD8 dose (data not shown). All the antibodies administered to mice were acquired from Bio X Cell, and were injected diluted in the buffer recommended by the vendor. Tumour size was assessed with a calliper by two researchers not blinded for the treatment group, and was calculated according to the formula: length × width^2^/2. Mice were euthanised with pentobarbital when tumours reached 1000 mm^3^. Complete tumour regression was defined as the absence of tumour, as assessed by palpation, by the end of a 60-days follow-up period after treatment initiation. Survival fractions for a given time point after treatment initiation were estimated according to the number of mice that have reached 1000 mm^3^ in relation to the total initial number of mice, using the Kaplan-Meier method. The statistical significance of the difference between curves was assessed using the Log-rank test. The synergistic interaction between atovaquone and aPD-L1 was determined by applying the Bliss independence model to the survival analysis, as previously described [11]. Briefly, a survival curve modelling the effect of the addition of the two treatments, assuming their effect is independent, was generated using the formula *S*_atovaquone/aPD-L1_ = 1 − (1 − *S*_atovaquone_ (*t*)) × (1 − *S*_aPD-L1_ (*t*)), where *S* are the corresponding survival fractions as a function of time (t). Then, we assessed the statistical significance of the difference between the *S*_atovaquone/aPD-L1_ and the atovaquone plus aPD-L1 curves by applying the Log-rank test.

### Re-challenge assay

Those mice treated with atovaquone plus aPD-L1 that showed complete regression 2 months after treatment initiation, along with naïve age-matched female Balb/c mice, were injected subcutaneously in the left flank with 3 × 10^5^ 4T1 or 4 × 10^5^ CT26 cells suspended in 30% Matrigel/PBS. Tumour development was assessed by palpation. Survival fractions for a given time point after injection of tumour cells were estimated according to the number of mice that have developed tumour in relation to the total initial number of mice, using the Kaplan-Meier method. Three weeks after tumour inoculation, the mice injected with CT26 cells were sacrificed, CD8+ cells were isolated from resected spleens using a MACS mouse CD8+ isolation kit (Miltenyi) following the manufacturer’s instructions. Then, CD8+ cells were suspended in RPMI/10%FBS and seeded in a U-bottom 96-well plate alone or in the presence of CT26 cells at a 1:3 ratio (CD8+:CT26), and 1 ng/mL IFNγ for MHCI induction. 24 h later, cells were labelled with Live/Dead stain (Thermofisher), and fluorophore-conjugated anti-mouse CD45 (clone 30-F11), CD8 (53-6.7), CD44 (IM7) and PD-1 (29f.1a12; all from BioLegend), and analysed with an Attune NxT cytometer (ThermoFisher). The percentage of live CD45 + /CD8+ cells positive for CD44 and PD-1 was assessed using a positivity threshold based on the corresponding FMO controls, and sequentially gating cells negative for Live/Dead staining, then CD45+ cells, CD8+ cells and, finally, either CD44+ or PD-1+ cells.

### Analysis of tumour-infiltrating CD8+ cells

Mouse tumours were resected, minced with a scalpel, and digested with 0.05% collagenase A (Sigma) in HBSS buffer for 30 min at 37 ^o^C. Cells were passed through a 40 µm strainer and incubated with red blood cell lysis buffer (BioLegend) for 5 min at room temperature. Cells were subsequently labelled with Live/Dead stain (Thermofisher), antibodies against CD45 (clone 30-F11), CD8 (53-6.7), and PD-1 (29f.1a12; all from BioLegend), and finally analysed by flow cytometry as described above for the re-challenge assay.

### Analysis of haematological and biochemical parameters in blood

Sixteen days after treatment initiation, blood was sampled by cardiac puncture under terminal anaesthesia induced with 140 mg/kg pentobarbital. For the analysis of haematological parameters, blood was collected into EDTA-coated tubes and kept on ice until analysis. For the biochemical parameters, blood was left to clot at room temperature and centrifuged at 2 × 10^3 ^*g* for 15 min at 4 ^o^C. Serum was collected from the supernatant and frozen on dry ice until analysis. The biochemical and haematological analyses were performed by MRC Harwell (UK) using a AU680 and an Advia 2120 analyser, respectively.

### Statistics

Two-tailed t-tests were used to calculate statistical significance unless indicated otherwise. A *p*-value of <0.05 was considered as statistically significant.

## Results

**Fig. 1:**
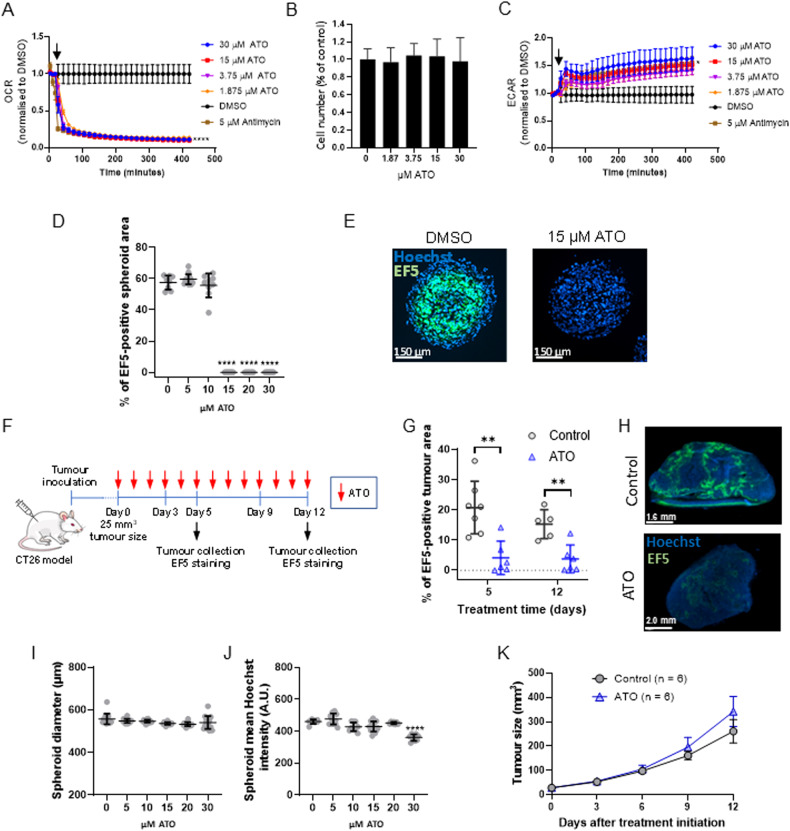
Atovaquone inhibits oxygen consumption and alleviates hypoxia in the CT26 cancer cell line in vitro and in vivo. **A** OCR of CT26 cells before and after (indicated by the arrow) the addition of the indicated concentrations of atovaquone (ATO). Antimycin A was used as a positive control of OCR inhibition. **B** Cell number from experiment described in (**A**), assessed in cells fixed just after the last OCR measurement (about 3 h after atovaquone addition). **C** ECAR assessment in CT26 cells before and after (indicated by the arrow) the addition of the indicated concentrations of ATO. Antimycin A was used as a positive control of ECAR induction. **D** CT26 spheroids were treated with the indicated concentrations of ATO for 24 h and incubated with the hypoxia probe EF5. Then, spheroids were processed for fluorescence microscopy analysis to assess the % of EF5-positive area in relation to the total spheroid area as determined by Hoechst staining. **E** Representative fluorescence microscopy images from (**D**). **F** Diagram of the experimental setup corresponding to the results shown in (**G**), (**H**) and (**K**). **G** CT26 tumour-bearing mice were treated with either ATO (200 mg/kg/day) or vehicle (Control) for 5 and 12 consecutive days. Then mice were injected with EF5 and tumours were resected and processed for fluorescence microscopy. The graph shows the % of EF5-positive area in relation to the total tumour area. **H** Examples of fluorescence microscopy images from (**G**) (day 12). **I** Diameters and (**J**) average Hoechst staining intensity of spheroids from (**D**), assessed in spheroids fixed 24 h after treatment initiation (A.U.: arbitrary units). **K** Tumour size of mice treated as described in (**F**) and (**G**) (average ± standard error; no statistically significant differences were found (ANOVA)). The dots in (**D**), (**G**), (**I**) and (**J**) represent individual spheroids/tumours. Data shown in (**A**–**D**), (**G**), (**I**) and (**J**) correspond to average ± standard deviation from a representative experiment repeated at least three times. *p* value: Student’s *t* test (*p < 0.05; **p < 0.005; ****p < 0.0001).

To assess the therapeutic efficacy of anti-PD-L1 in combination with atovaquone, we used the immunocompetent CT26 syngeneic model of colorectal cancer. Before starting the in vivo experiments, we tested the capacity of atovaquone to inhibit the basal oxygen consumption rate (OCR) in CT26 cells. As shown in Fig. 1A, atovaquone is able to decrease the basal OCR of CT26 cells in vitro –like the Complex III inhibitor antimycin A used as positive control– in a dose-dependent fashion. The reduction in OCR with atovaquone was not associated with a decrease in cell number, confirming that atovaquone efficiently targets the ETC in these cells (Fig. 1B). Furthermore, the analysis of the extracellular acidification rate (ECAR), indicative of the aerobic glycolysis rate, revealed an increase with atovaquone treatment (Fig. 1C). This suggests that CT26 cells compensate for the loss of energy production via OXPHOS by enhancing the glycolytic metabolism.

Next, we tested whether atovaquone was able to reduce the hypoxic core that forms in multicellular spheroids derived from CT26 cells cultured in vitro and which can be visualised using the hypoxia probe EF5. Incubation of CT26 spheroids with atovaquone resulted in the complete disappearance of the hypoxic core at concentrations greater than 15 µM atovaquone (Fig. 1D, E). Likewise, oral administration of atovaquone to CT26 tumour-bearing mice led to a significant and stable reduction in tumour hypoxia, as demonstrated by a decrease in EF5 staining (Fig. 1F–H). We determined the spheroid size (Fig. 1I), the average intensity of the nuclei staining as a readout of the cell density in the spheroid (Fig. 1J), as well as the tumour size (Fig. 1K), to capture potential differences in tumour cell viability or proliferation due to the treatment with atovaquone. Except for a slight decrease in cell density in spheroids treated with 30 µM atovaquone, no significant differences were found in any of these parameters with atovaquone treatment. This suggests that atovaquone alleviates hypoxia primarily through inhibition of oxygen consumption rather than by decreasing cell viability or proliferation, in line with our previous in vitro and in vivo studies in human cancer models [8, 9].

**Fig. 2:**
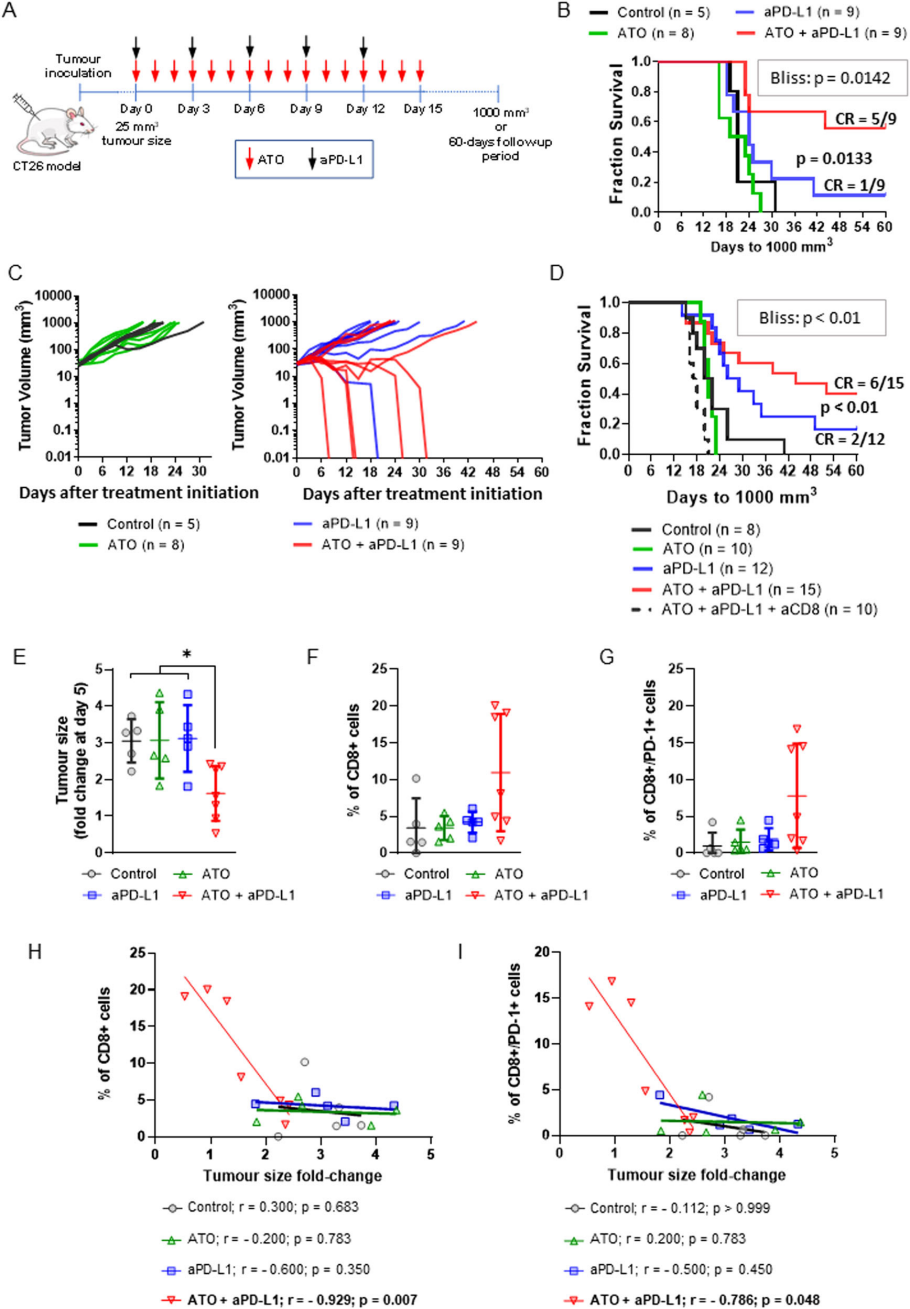
Atovaquone enhances the efficacy of aPD-L1 treatment in a CD8+ cell-dependent manner. **A** Diagram of the experimental setup corresponding to the results shown in (**B**) and (**C**). **B** Survival analysis and (**C**) individual tumour growth graphs from BALB/c mice bearing subcutaneous CT26 tumours treated with ATO (200 mg/kg/day for 15 days by gavage) and/or aPD-L1 (a total of 5 × 10 mg/kg i.p. injections, administered every 3 days), starting at a tumour size of about 25 mm^3^. The proportions of mice experiencing complete tumour regression (CR) are indicated in (**B**). **D** Mice bearing CT26 tumours were essentially treated as in (**A**–**C**) but including a group treated with anti-CD8α (aCD8; 400 µg on day -2 and day 1, and 200 µg every 6 days thereafter). ATO and/or aPD-L1 treatments were started at a tumour size of about 40 mm^3^. Curve comparisons in (**B**) and (**D**): Log-rank test; the p values corresponding to the Bliss independence test for the synergy between ATO and aPD-L1 are also provided (Bliss). **E**–**I** BALB/c mice bearing subcutaneous CT26 tumours were treated as in (**C**) (Control, *n* = 5; ATO = 5; aPD-L1 = 5; ATO + aPD-L1 = 7). Tumours were collected at day 5 and analysed by FACS to determine the % of total and activated (PD-1+) infiltrating CD8+ T cells. **E** Fold-change in tumour size at day 5 in relation to the tumour size at treament start. Graphs show the % of total CD8+ (**F**) and CD8+/PD-1+ (**G**) cells in relation to CD3+ cells. % of CD8+ (**H**) and CD8+/PD-1+ (**I**) cells plotted against fold-change in tumour size at day 5. Lines represent linear regression curves. Spearman’s correlation coefficients (*r*) and the corresponding *p* values are provided below the graphs. The dots in (**E**–**I**) represent individual tumours. Data shown in (**E**–**G**) correnspond to average ± standard deviation (unless otherwise indicated, no statistically significant differences were found; *p < 0.05, ANOVA).

Having shown that atovaquone effectively inhibits oxygen consumption and alleviates hypoxia in the CT26 model, we next assessed the anti-tumour efficacy of aPD-L1 in combination with atovaquone in an in vivo tumour growth delay assay (Fig. 2A–D). Mice treated with atovaquone alone and control mice displayed similar tumour growth. In contrast, the group treated with atovaquone plus aPD-L1 showed a slower tumour growth rate and a higher proportion of mice with complete tumour regression than the group treated with aPD-L1 alone. The Bliss test for synergy demonstrated a statistically significant synergistic effect between atovaquone and aPD-L1 (Fig. 2B, D).

We next asked whether atovaquone potentiates the anti-tumour effect of aPD-L1 through an immune-mediated mechanism. It is broadly accepted that cytotoxic CD8+ lymphocytes are the ultimate mediators of the anti-tumour immune response unleashed by ICB. Depleting CD8+ T lymphocytes completely abrogated the effect of the combination treatment (Fig. 2D). Furthermore, we determined the proportion of both total and activated/PD-1+ [12] CD8+ T cells in the tumour at day 5 after treatment initiation, when tumours from mice treated with ATO plus aPD-L1 already display a significant tumour size decrease as compared with the other treatments, but no tumour has experienced complete regression yet (Fig. 2E). At that time point, both the average proportion of total CD8+ and CD8+/PD-1+ T cells tended to be higher in the group of mice treated with ATO plus aPD-L1, although these differences were not statistically significant (Fig. 2F, G). However, we found a strong negative correlation between the tumour levels of total/PD-1+ CD8+ T cells and tumour size specifically in the ATO/PD-L1-treated group, which reflects the activation of a CD8+ T cell-mediated response in those tumours with delayed growth (Fig. 2H, I). Altogether, these results suggest that the addition of atovaquone to aPD-L1 promotes the early activation of CD8+ cells and an anti-tumour immune response that is dependent on T cell-mediated killing.

**Fig. 3:**
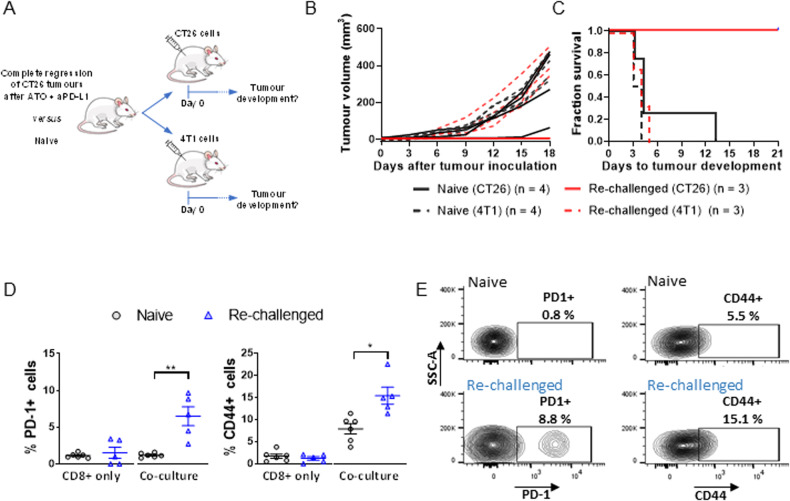
Tumour eradication in CT26 tumour-bearing mice treated with atovaquone and aPD-L1 is linked to the development of a memory response. **A** Schematic representation of the experimental setup corresponding to the results shown in (**B**) and (**C**). **B**, **C** Mice previously inoculated with CT26 tumours and treated with ATO + aPD-L1 from experiment described in Fig. 2D showing complete regression 2 months after treatment initiation (“Re-challenged”) and naïve mice were injected subcutaneously with either CT26 (*n* = 3) or 4T1 (breast, *n* = 3) tumour cells and monitored for tumour development. **D** Mice previously inoculated with CT26 tumours and treated with ATO + aPD-L1 which showed complete tumour regression 2 months after treatment initiation (“Re-challenged”; from Fig. 2B, C) and naïve mice were inoculated with CT26 cells and sacrificed 21 days later. Their splenic CD8+ cells were then isolated and co-cultured in vitro with (“co-culture”) or without (“CD8+ only”) CT26 cells for 24 h and analysed by FACS for PD-1 and CD44 surface expression. *p < 0.05; **p < 0.005 (Student’s *t* test). **E** Examples of FACS contour plots from experiment described in (**D**).

We also demonstrated that mice with complete tumour regression after atovaquone and aPD-L1 combination treatment do not develop tumours after re-challenge with CT26 cells, unlike those challenged with a different syngeneic cancer cell line (4T1) (Fig. 3A–C). This indicates that the treatment with atovaquone plus aPD-L1 results in the establishment of a durable, tumour-specific anti-tumour immune response in those mice with complete remission. Consistent with the development of a CD8+ T cell-mediated memory response, splenic CD8+ cells from mice treated with atovaquone plus aPD-L1 experiencing durable tumour eradication showed increased levels of T cell activation markers (CD44, PD-1) when challenged ex vivo with CT26 cells (Fig. 3D, E). Altogether, these results suggest that atovaquone favours the development of a tumour-specific memory immune response upon ICB.

**Fig. 4:**
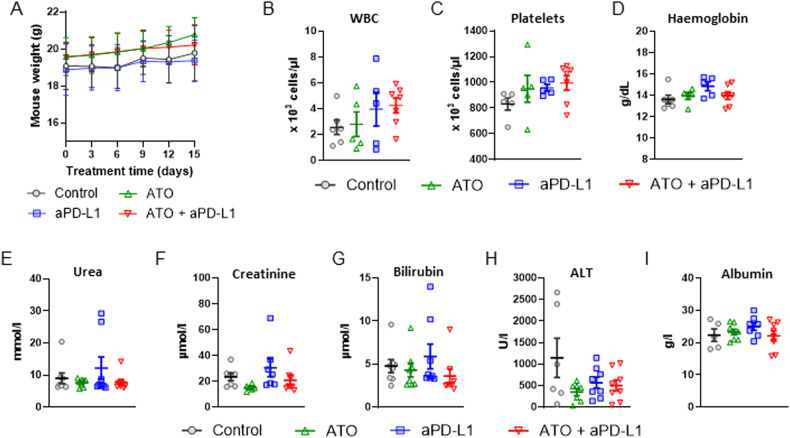
Treatment with atovaquone and aPD-L1 does not cause toxicity. BALB/c mice bearing subcutaneous CT26 tumours treated with ATO (200 mg/kg/day for 15 days by gavage) and/or anti-PD-L1 (a total of 5 × 10 mg/kg i.p. injections, administered every 3 days) starting at a tumour size of about 25 mm^3^. Mouse weight was recorded throughout the experiment (**A**). One day after the end of the treatment, blood was sampled by cardiac puncture under terminal anaesthesia, and haematological (**B**–**D**) and plasma clinical chemistry (**E**–**I**) parameters were measured. Mean values ± standard errors are shown. WBC White blood cells, ALT alanine aminotransferase. No statistically significant differences were found (ANOVA).

Mouse weight analysis revealed no weight loss indicative of toxicity with any of the treatments (Fig. 4A). We additionally analysed a panel of haematological and biochemical plasma markers. Haematological markers included white blood cell count (Fig. 4B), platelet count (Fig. 4C) and haemoglobin (Fig. 4D). Biochemical parameters included markers of renal (urea, creatinine; Fig. 4E, F) and hepatic toxicity (bilirubin, alanine aminotransferase and albumin; Fig. 4G, I). None of the treatments caused significant differences in the levels of these markers, indicating that the synergy between atovaquone and aPD-L1 is not associated with any toxicity.

**Fig. 5:**
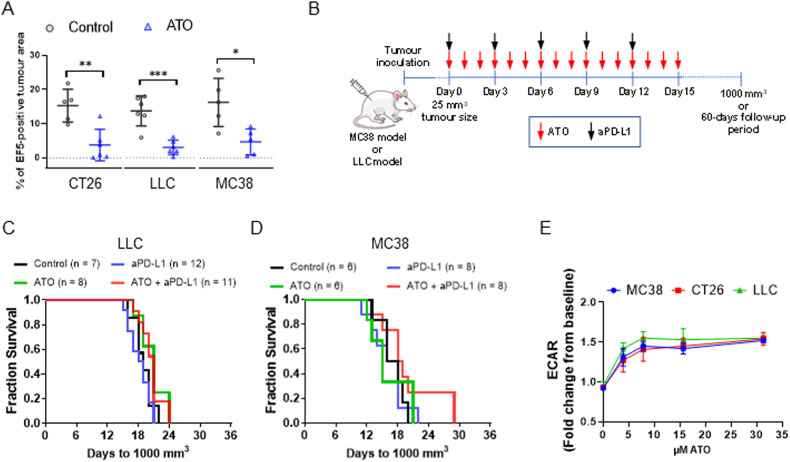
Atovaquone does not enhance the efficacy of aPD-L1 treatment in the LLC and MC38 syngeneic mouse models. **A** % of EF5-positive area in tumours from mice bearing subcutaneous CT26, LLC or MC38 tumours and treated with either CMC (Control) or 200 mg/kg/day ATO for 9 days. Mean ± standard deviation. *p < 0.05; **p < 0.005; ***p < 0.001 (Student’s *t* test). **B** Schematic representation of the experimental setup corresponding to the results shown in (**C**) and (**D**). **C**, **D** Survival analysis of C57BL/6 mice bearing subcutaneous LLC or MC38 tumours treated with ATO (200 mg/kg/day for 15 days by gavage) and/or aPD-L1 (a total of 5 × 10 mg/kg i.p. injections, administered every 3 days), starting at a tumour size of about 25 mm^3^. No statistically significant differences were found (log-rank test). **E** Extracellular acidification rate (ECAR) of CT26, LLC and MC38 cells treated with the indicated concentrations of ATO. Fold induction values were calculated by dividing the baseline ECAR by the ECAR values obtained 2 h after atovaquone addition. Data correspond to mean ± standard deviation (*n* = 3 technical replicates; representative from an experiment repeated two times).

Finally, to see whether atovaquone synergised with anti-PD-L1 in other models with a different responsiveness to aPD-L1 treatment than the CT26 model, we carried out tumour growth delay experiments in C57/BL6 mice bearing MC38 (colorectal) and LLC (lung) tumours. These models were selected because they have a significant extent of tumour hypoxia (Fig. 5A), and have been previously shown to be less responsive than CT26 tumours to the same aPD-L1 antibody clone used in the present study, using similar treatment schedules [13, 14]. As shown in Fig. 4A, atovaquone treatment significantly decreased hypoxia in both MC38 and LLC tumours, at levels comparable to those achieved in CT26 tumours. However, atovaquone failed to synergise with aPD-L1 treatment in these models (Fig. 5B–D). The acidification of the tumour microenvironment due to the high glycolytic metabolism of tumour cells has been shown to suppress the anti-tumour immunity [15, 16]. Since upon ETC inhibition cells activate aerobic glycolysis to keep adequate ATP levels, we asked whether the model-dependent response to atovaquone plus aPD-L1 could be explained by a greater induction of aerobic glycolysis in the MC38 and LLC models. The analysis of the ECAR in vitro revealed no differences in the magnitude of induction of this parameter with atovaquone treatment across the MC38, LLC and CT26 cell lines (Fig. 5E). This suggests that a greater acidification of the tumour microenvironment by atovaquone is unlikely to be a major factor in the lack of response to atovaquone and aPD-L1 in the MC38 and LLC models.

Overall, our results suggest that atovaquone can safely enhance the anti-tumour effect of aPD-L1, but the efficacy of this combination treatment seems to rely on the tumour cell model. Therefore, more studies are needed to determine the mechanisms limiting this synergistic effect and, ultimately, to find predictive biomarkers that enable the identification of patients more likely to respond to this combination treatment.

## Discussion

Our group recently showed that inhibiting mitochondrial complex III with atovaquone is an effective and safe strategy to alleviate hypoxia in cancer patients [9, 10]. This finding may have significant implications in cancer therapy, as tumour hypoxia is a key factor limiting the efficacy of cancer treatments and, so far, therapeutic strategies specifically aimed at reducing tumour hypoxia have not been implemented into widespread clinical practice. In the present study, we demonstrate that atovaquone potentiates the effect of aPD-L1 therapy linked to its ability to alleviate tumour hypoxia in the CT26 murine model of colorectal cancer, by favouring a durable anti-tumour immune response and without causing any toxicity. However, this synergistic effect was not paralleled in the other two syngeneic models investigated –the LLC and MC38 models– despite the levels of basal hypoxia and the degree of hypoxia alleviation with atovaquone treatment being similar to those observed in CT26 tumours. Therefore, more studies are needed to clarify what factors restrict the synergy between atovaquone and aPD-L1 before testing this combination treatment in patients.

Our observations raise the question as to whether hypoxia exerts an immunosuppressive role in the models tested in the present study and, particularly, whether atovaquone affects the response to aPD-L1 through a hypoxia-dependent mechanism in the CT26 model. To shed light on whether the synergy between atovaquone and aPD-L1 relies on hypoxia alleviation, it would be interesting to assess whether there is an influx of immune cells (e.g., CD8+ cells) to areas that become oxygenated upon atovaquone treatment. However, there are currently no techniques that allow to readily monitor the dynamics of tumour hypoxia on fixed tissue sections to delineate the tumour areas that become oxygenated [17].

The immunosuppressive role of hypoxia is thought to be exerted through different mechanisms of action, orchestrated through a network of soluble mediators and immune cells. Some of these mechanisms depend on the direct sensing of low oxygen levels via the hypoxia-inducible factor (HIF) pathway (e.g., HIF-mediated inhibition of effector T cell activity) [18, 19], while others rely on the effect of oxygen on cell metabolism [16, 18, 20]. In this regard, upon limited oxygen levels, cells cannot efficiently produce energy via OXPHOS and, as a compensatory mechanism, increase aerobic glycolysis, which leads to the acidification of the tumour microenvironment [20]. OXPHOS inhibition, glycolysis and the acidic microenvironment can inhibit the anti-tumour immune response at different levels (e.g., by abrogating immunosurveillance by T and natural killer cells, or favouring infiltration of myeloid-derived suppressor cells) [15, 16, 18–20]. It is important to note that atovaquone increases oxygen availability by inhibiting OXPHOS, and this may lead to the induction of aerobic glycolysis and acidification of the tumour microenvironment [21]. Therefore, although atovaquone may relieve some oxygen-sensing-dependent immunosuppressive mechanisms, it is not expected to diminish, or could even exacerbate immunosuppressive mechanisms via direct OXPHOS inhibition, induction of glycolytic metabolism and acidification of the tumour microenvironment. According to our in vitro experiments, LLC, MC38 and CT26 tumour cells do not display differences in the magnitude of the glycolytic shift induced by atovaquone, so we believe that the acidification of the tumour microenvironment is not a major factor that explains the model-dependent effect of atovaquone on the response to aPD-L1, although this should be confirmed in vivo.

A distinctive feature between the CT26 and the LLC and MC38 models that may explain the differential response to atovaquone and aPD-L1 treatment is the tumour immune profile. While LLC and MC38 tumours are rich in immunosuppressive immune cells –particularly myeloid-derived suppressor cells– CT26 tumours display an immunogenic phenotype with a more equitable proportion of cytotoxic effector and immunosuppressive cells, which correlates with a higher ICB efficacy [13, 14]. Importantly, tumour expression of PD-L1 positively correlates with the response to aPD-L1 therapy and, in turn, high PD-L1 expression in tumours has been linked to an immunogenic tumour microenvironment [22, 23]. In line with this, it has been shown that the MC38 model displays lower tumour PD-L1 levels than the CT26 model [24, 25]. Therefore, the presence of a cytotoxic cell–rich tumour immune infiltrate may not only be a major determinant of the response to aPD-L1 treatment alone, but also of the synergistic effect between atovaquone and aPD-L1. Accordingly, the existence of tumour cell-intrinsic, hypoxia-independent mechanisms that limit the development of an effective anti-tumour immune response, and which would predominate over the mechanisms of hypoxia-mediated immunosuppression, could underly both the limited response to aPD-L1 and the lack of synergism between atovaquone and aPD-L1 in the LLC and MC38 models. One of these potential mechanisms of immunosuppression could be, for example, the secretion of immune-suppressive cytokines by tumour cells [26, 27]. In summary, the immune cell composition of the tumour and known tumour-intrinsic immunosuppressive mechanisms should be considered in future research aimed at identifying markers of response to atovaquone and aPD-L1.

Other ETC inhibitors have been previously tested in preclinical models in combination with ICB. The mitochondrial Complex I inhibitor metformin, a drug commonly used to treat type 2 diabetes, potentiates the anti-tumour effect of aPD-1 and aCTLA4 in murine models [28–30]. Scharping et al. [29] reported a therapeutic benefit when combining aPD-1 and metformin in the MC38 model, which somehow contrasts with the lack of benefit reported in the present study when adding atovaquone to aPD-L1 in this model. This apparent discrepancy may be explained by reported differences in the effects of aPD-L1 and aPD-1 on the anti-tumour immune response [31, 32]; and/or by the purported diversity of targets metformin can interact with [33, 34], and which may therefore exert an effect on the response to ICB independent of its capacity to inhibit the ETC. Notwithstanding the preclinical evidence, retrospective studies have failed to show significant improvement in the outcomes of patients treated with metformin in combination with aPD-L1 (atezolizumab), aPD-1 (nivolumab, pembrolizumab) and/or aCTLA4 (ipilimumab) [35–38]. Metformin has also failed to improve the clinical outcomes of non-small cell lung cancer patients treated with chemo-radiotherapy [39]. The absence of patient selection based on the levels of tumour hypoxia may partly explain the lack of therapeutic benefit of metformin in these clinical studies.

Although aPD-1 and aPD-L1 antibodies target the same ICB axis, differences in the immune response triggered by these therapies have been reported [31, 32]. It would therefore be interesting to test whether the efficacy profile of atovaquone plus aPD-1 resembles the one reported here for atovaquone plus aPD-L1. It would also be interesting to test atovaquone with aCTLA4 –the other major ICB used clinically– which, unlike aPD-1/PD-L1 therapies, primarily acts at the priming stage of T cell activation [40, 41]. In terms of the treatment administration schedule, in our study, atovaquone was administered at the start of aPD-L1 treatment to ensure the reduction in tumour hypoxia before and long after the reduction in tumour size with aPD-L1 treatment become apparent. Although changes in the immune system induced by ICB occur before any observable change in tumour size, the body often requires time to build an effective anti-tumour response, so the timing of administration of atovaquone in relation to ICB may deserve further evaluation in future studies.

In addition to its limiting effect on ICB efficacy, the most direct and well-known implication that hypoxia has in cancer treatment is that it reduces the sensitivity of tumours to radiotherapy [42]. While DNA lesions caused by ionising radiation become ‘fixed’ when molecular oxygen is present, under hypoxia these are quickly restored to its original state, which reduces cancer cell death and, consequently, the anti-tumour effect of radiotherapy [43]. Our group has previously demonstrated that hypoxia alleviation by atovaquone increases the sensitivity of tumour xenografts to radiation [8]. Importantly, both preclinical and clinical studies have demonstrated that radiotherapy augments the efficacy of ICB by impacting several immune processes, including the induction of immunogenic cell death, tumour antigen-specific T cell priming and recruitment of leucocytes into tumours [44, 45]. It can, therefore, be expected that atovaquone-mediated hypoxia inhibition will boost these radiotherapy-induced immune processes, which could be exploited to further increase the therapeutic benefit of ICB plus radiotherapy. Although the mitochondrial Complex I inhibitor IACS-010759 has been shown to enhance the anti-tumour effect of aPD-1 combined with radiotherapy in a mouse lung cancer model [46], a recent clinical trial has evidenced that IACS-010759 has substantial adverse effects [47]. In contrast, atovaquone has a very good and well-documented safety profile, warranting further studies with this ETC inhibitor in combination with ICB and radiotherapy.

## Data Availability

The information related to this article will be made available to readers upon request.
